# Hirudin in the Treatment of Chronic Kidney Disease

**DOI:** 10.3390/molecules29051029

**Published:** 2024-02-27

**Authors:** Sai-Ji Liu, Yi-Ling Cao, Chun Zhang

**Affiliations:** Department of Nephrology, Union Hospital, Tongji Medical College, Huazhong University of Science and Technology, Wuhan 430022, China; easonsj_2008@163.com (S.-J.L.); saintiker@foxmail.com (Y.-L.C.)

**Keywords:** chronic kidney disease, hirudin, renal fibrosis, inflammation

## Abstract

Chronic kidney disease (CKD) is a common public health concern. The global burden of CKD is increasing due to the high morbidity and mortality associated with it, indicating the shortcomings of therapeutic drugs at present. Renal fibrosis is the common pathology of CKD, which is characterized by glomerulosclerosis, renal tubular atrophy, and renal interstitial fibrosis. Natural hirudin is an active ingredient extracted from *Hirudo medicinalis*, which has been found to be the strongest natural specific inhibitor of thrombin. Evidence based on pharmacological data has shown that hirudin has important protective effects in CKD against diabetic nephrology, nephrotic syndrome, and renal interstitial fibrosis. The mechanisms of hirudin in treating CKD are mainly related to inhibiting the inflammatory response, preventing apoptosis of intrinsic renal cells, and inhibiting the interactions between thrombin and protease-activated receptors. In this review, we summarize the function and beneficial properties of hirudin for the treatment of CKD, and its underlying mechanisms.

## 1. Introduction

The kidney is a vital organ of the urinary system. Its physiological functions are mainly to excrete metabolites, regulate water, electrolytes, and acid–base balance to maintain the stability of the body’s internal environment, and endocrine function [1]. Chronic kidney disease (CKD) is defined as abnormalities of renal structure or function presenting for at least 3 months [2]. Diabetes, hypertension, and primary glomerular diseases are major contributors to CKD [3]. CKD is a progressive systemic disease that manifests as renal fibrosis, which causes irreversible damage to renal structure and function over months and years, leading to renal failure. The most used international diagnostic criteria are estimated glomerular filtration rate (eGFR) < 60 mL/min/1.73 m^2^ or urinary albumin-to-creatinine ratio (ACR) ≥ 30 mg/g. The burden of CKD is substantial. The global prevalence of CKD is estimated to be 10–15% [4,5,6], with CKD predicted to become the fifth most prevalent chronic condition by 2040 [7]. CKD is associated with adverse cardiovascular disease outcomes, and gradually develops into end-stage renal disease (ESRD) requiring dialysis or transplantation [8,9]. CKD is estimated to impact more than 700 million people worldwide, leading to physical restrictions, low quality of life, mental illness, social isolation, and premature death [10,11]. The treatment of CKD still has no specific method, and mainly involves controlling protein and salt intake, blood pressure, blood glucose, blood lipids, and other risk factors [12,13]. Angiotensin-converting enzyme inhibitors or angiotensin II receptor blockers are usually used as first-line drugs to delay the progression of CKD [14]. However, some patients exhibit insufficient response to renin–angiotensin system inhibitors, or experience severe side effects from such medications including hyperkalemia and worsening of renal function, indicating the shortcomings of therapeutic medicines at present. These urgent problems have prompted researchers to find more drugs and methods for the treatment of CKD.

Since ancient times, many natural products have been discovered and become valuable medicines [15]. The constituent compounds of natural products receive considerable attention in the research of kidney disease both in vivo and in vitro [16]. *Hirudo medicinalis*, belonging to hematophagous leeches, first recorded in *Shennong’s Classic of Material Medical*, has been used for medical treatment since ancient times. In traditional Chinese medicine, the leech has the effect of breaking blood, dissipating blood stasis, and dredging collaterals [17]. It has been reported that leeches may improve renal function and reduce the occurrence of proteinuria in diabetic nephrology rats [18]. Natural hirudin is an active ingredient extracted from *Hirudo medicinalis* and its salivary glands, which has been found to be one of the most potent and specific natural inhibitors of thrombin [19,20]. Based on its potent antithrombotic properties, hirudin has been used to treat some thrombotic disorders like coronary artery disease and deep vein thrombosis [21,22]. Meanwhile, other biological functions of hirudin have been gradually discovered, including wound repair [23,24], anti-tumor [25,26], anti-ischemic effects [27], and anti-pulmonary fibrosis [28]. Furthermore, hirudin has been used in cellular or animal models of CKD for different types of research. The results suggest that hirudin may have preventive effects in vitro or in vivo, offering a potential treatment for the disease. This article systematically reviews the role and beneficial effects of hirudin in CKD.

## 2. Pathophysiology of CKD

Renal fibrosis is the common pathology of CKD. The main processes of the initial and progression of renal fibrosis are nephron loss, inflammation, myofibroblast activation, and extracellular matrix (ECM) deposition [29,30], which ultimately lead to renal failure and ESRD [31]. Renal fibrosis refers to the unsuccessful wound healing of kidney tissue after chronic and persistent injury such as inflammation or immune-mediated or toxic injury, and is characterized by glomerulosclerosis, renal tubular atrophy, and renal interstitial fibrosis (RIF) [3].

Glomerulosclerosis is caused by glomerular endothelial injury and dysfunction, abnormal proliferation of smooth muscle cells and mesangial cells, loss of podocyte integrity, and dysregulation of cellular metabolism [32,33,34,35]. Glomerular endothelial cells (GEC) respond to hypertension to activate glomerular microinflammation, which in turn activates mesangial cell proliferation and ECM accumulation such as fibronectin and collagens [36]. Meanwhile, stretching of the podocytes exposes areas of the glomerular basement membrane to the Bowman’s capsule and forms adhesions with it, leading to glomerulosclerosis [37]. During renal injury, renal tubular epithelial cells (RTEC) transform into a secretory phenotype, which synthesizes reactive oxygen species (ROS) and proinflammatory cytokines, such as tumor necrosis factor-α (TNF-α) and interleukin-1β (IL-1β) [38]. These agents aggravate inflammatory cell infiltration and induce interactions with interstitial myofibroblasts. Injured tubular epithelia undergo apoptosis and lose their primary space, which leads to tubular atrophy [39,40]. If the injury is repeated or severe, ECM proteins continue to accumulate in the kidneys, leading to tissue destruction, kidney dysfunction, and eventually organ failure [41].

## 3. Hirudin

### 3.1. Structure and Derivatives of Hirudin

**Figure 1:**
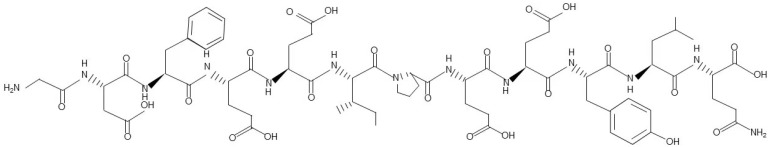
The chemical structure of natural hirudin.

Hirudin was discovered in 1884 by Haycraft when he found that leech extract had anticoagulant properties; then, the extract was officially named hirudin in 1904 by Jacoby. The relatively pure hirudin was first isolated from the salivary glands of *Hirudo medicinalis* in 1955 by Markward [42]. The complete amino acid sequence of hirudin was first described in 1984 by Dodt. Hirudin is a single-chain polypeptide of 65 amino acids that consists of a compressed N-terminal region (amino acids 1–47) cross-linked by three disulfide bonds and a flexible C-terminal tail with a negative charge [43]. The chemical structure of natural hirudin is shown in Figure 1. From then on, more than ten diverse hirudin variants (HV) have been found in leeches. HVs have been reported to be highly active and stable, binding thrombin with an association constant as high as 4.7 × 108 mol^−1^ S^−1^, and having a dissociation constant as low as 2.3 × 10^−14^ mol. Therefore, the complex of hirudin and thrombin is stable, and can delay the degradation of fibrinogen and the reaction between platelets [44]. However, the low yield and high cost of natural hirudin limit its application and promotion. The development of bioengineering technology has solved this problem, and recombinant hirudin (rH) has been synthesized through genetic engineering. The rH has similar pharmacological activity and chemical structure compared to natural hirudin [45]. Moreover, to reduce the side effects of hirudin and improve its bioavailability, more hirudin derivatives have been synthesized and improved. Some derivatives have been widely used for clinical treatment in thrombotic diseases including lepirudin, desirudin, and bivalirudin [46,47,48].

### 3.2. Isolation Procedures of Hirudin

In 1904, Jacoby, a German pharmacologist named the term “hirudin” to refer to the leech head extract produced through a patented purification technology [49], which started the commercial production of hirudin. The emergence of commercial hirudin preparations has encouraged pharmacological research on its mechanism of action.

In early times, researchers used organic solvents or aqueous mixtures of organic solvents for extraction and precipitation. The remaining contaminants were separated by paper electrophoresis, during which the antithrombin activity of hirudin was used to monitor the isolation and purification steps [50].

In subsequent years, various purification methods emerged based on chromatographic principles. Gel filtration facilitated the separation of both high- and low-molecular-weight contaminants from the thrombin inhibitor. Under neutral pH conditions, hirudin was anticipated to elute as a dimer, while at pH 3.0, it was expected to elute as a monomer. Additional contaminations were separated through chromatography using substances such as Amberlite IRC 50, CM-Sephadex, diethylaminoethyl (DEAE)-Sephadex, or DEAE-Sephacryl. Hirudin elution was achieved by augmenting the sodium chloride concentration or by reducing the pH of the buffer. Among other constituents, crude hirudin preparations contained trypsin-plasmin inhibitors, which could be isolated from hirudin through affinity chromatography on trypsin-Sepharose.

Similar to the formation of complexes between thrombin and inhibitors in solution, hirudin adhered to thrombin-Sepharose and underwent isolation through affinity chromatography. Elution was facilitated using the low-molecular-weight inhibitor benzamidine or its derivatives. Subsequently, hirudin underwent separation through gel filtration on Sephadex G25 immediately after elution with benzamidine in a sequential procedure.

Additionally, hirudin formed complexes with thrombin, and this complex was distinguished from free thrombin via gel filtration on Sephadex G75. Within this complex, thrombin underwent inactivation through heating at 80 °C for 15 min. Hirudin with elevated specific activity was further isolated via gel filtration on Sephadex G75. Isoelectric focusing also offered a means to isolate hirudin from crude preparations.

In contemporary practices, the following isolation procedures are commonly employed: initial concentration of the extract through lyophilization or ultrafiltration, followed by crude separation via gel filtration and anion exchange chromatography, along with affinity chromatography on immobilized thrombin. The final stage of separation involves high-performance liquid chromatography (HPLC), a method capable of distinguishing different hirudin variants [51].

### 3.3. Pharmacokinetics of Hirudin

Oral absorption of effective concentrations is not possible due to the polypeptide structure of hirudin. Intestinal absorption of hirudin in experimental animals is very low. Very small amounts of hirudin were detected in urine (<0.1% oral absorption) only after oral administration in dogs. Therefore, hirudin can only be administered parenterally in humans [52]. Pharmacokinetic studies confirmed that hirudin distributes into the extracellular space throughout the body. Following intravenous administration, the elimination half-life of hirudin was approximately 1 h. Extravascular routes of administration extend this time to 2 to 3 h. Hirudin was eliminated completely by the kidneys and partly recovered unchanged in the urine. There were obvious species differences in hirudin excretion. Rats excreted only a small amount of active hirudin, while dogs almost completely excreted active hirudin. After human administration of hirudin (0.1 mg/kg), only about one-third of the dose is excreted in the urine [53].

### 3.4. Toxicology of Hirudin

Several studies in rodents and mammals tested the acute toxicity of hirudin. No systemic or organ-specific toxicities were detected at doses up to 300 mg/kg intravenously. Mucosal hemorrhages including pleura, pia mater, and peritoneum occurred in rats 8 days after subcutaneous injection of 100 mg/kg hirudin. Sub-chronic toxicity studies showed that daily doses of 1 to 5 mg/kg led to a dose-dependent increase in bleeding tendency in dogs and rats for up to 3 months. There is no evidence demonstrating the organ-specific changes in organ toxicity of hirudin. In dogs, antibodies are formed when very high doses of hirudin are given. It is important to note that these antibodies do not neutralize the antithrombin activity of hirudin, but bind it without impairing its action [54]. Such antibodies are also present in humans taking hirudin over a long period. In the initial study, up to 50% of patients produced low levels of hirudin antibodies. However, these antibodies do not precipitate or neutralize the functions of hirudin, but rather act as a reservoir of hirudin. Hirudin bound to the antibody continues to block thrombin generation [55]. The therapeutic range of hirudin’s antithrombotic effect in blood is 0.5 to 1.5 µg/mL. At a blood concentration of 2 µg/mL, hirudin led to an increased tendency to bleed as a toxic side effect. At higher blood levels, severe bleeding complications could occur, and antidotes or detoxification agents may be required [53]. Due to the narrow therapeutic window of hirudin, meaning the limited range between therapeutic and toxic doses, drug monitoring of hirudin is absolutely necessary.

### 3.5. Clinical Use of Hirudin Derivatives

Recombinant hirudin comes in two forms: lepirudin and desirudin. The two forms have similar structures except for their N-terminal sequences, namely Leu^1^-Tyr^2^ in lepirudin and Val^1^-Val^2^ in desirudin. Several randomized controlled trials have been conducted with recombinant hirudin in the prevention of venous thrombosis and in the treatment of severe myocardial ischemic diseases such as acute coronary syndromes (ACS). Desirudin was superior to enoxaparin in preventing proximal deep-vein thrombosis in patients after total hip replacement surgery [56]. For patients with unstable angina (UA) or non-ST-segment elevation acute myocardial infarction, lepirudin was more effective than heparin in preventing refractory angina, new myocardial infarction, and death. Desirudin also showed a curative effect for preventing major cardiovascular events in patients with ACS, but induced an increased risk of bleeding [57]. Compared to historical controls, lepirudin significantly reduced the risk of death and new thromboembolic events in patients with heparin-induced thrombocytopenia (HIT) and associated arterial and/or venous thrombosis [58]. Recombinant hirudin is currently approved for two indications, lepirudin for HIT complicated by thrombosis, and desirudin for thrombosis prophylaxis after major orthopedic surgery [56,59], as shown in Table 1.

Bivalirudin, a new synthetic hirudin, has a lower specific activity and a shorter half-period than hirudin [60]. Bivalirudin has been widely tested in patients with ACS and those undergoing PCI [61]. As an alternative to heparin, bivalirudin demonstrated noninferiority or superiority with a reduction in major bleeding, and did not cause thrombocytopenia. For patients with ST-segment elevation myocardial infarction undergoing PCI, bivalirudin could reduce all-cause mortality in the HORIZONS-AMI trial [62]. Consequently, bivalirudin has been widely used in the United States for PCI. Bivalirudin also showed efficacy and safety in patients with HIT antibodies requiring PCI or coronary bypass surgery [63]. Bivalirudin has been approved for patients with UA undergoing percutaneous transluminal angioplasty (PTCA), for those needing PCI with temporary use of glycoprotein inhibitor (GPI), and for patients requiring PCI who are at risk of HIT (Table 1) [57]. Recently, bivalirudin has become the primary anticoagulant alternative to unfractionated heparin (UFH) for extracorporeal membrane oxygenation (ECMO) support. Compared with UFH, bivalirudin reduced the risk of thrombosis, severe bleeding, and the need for transfusions in patients receiving ECMO [64,65].

## 4. Hirudin in CKD

### 4.1. Hirudin in Diabetic Nephrology

Diabetic nephropathy (DN) is a common and fatal microvascular complication of diabetes and a major cause of ESRD [66]. DN is characterized by diabetic glomerulopathy, the presence of pathological albumin in the urine, and decreased glomerular filtration rate (GDR) in diabetic patients [67]. Fibrosis, inflammation, oxidative stress, and apoptosis have been shown to play critical roles in the pathogenesis of DKD [68,69,70]. Various studies have explored the effect of hirudin in DN, and most of them showed a positive effect on renal function (Table 2).

Using network pharmacology and molecular docking to reveal the mechanism of hirudin in the treatment of DN, the results indicated hirudin may regulate various signaling pathways including the phosphatidylinositol 3-kinase (PI3K)-protein kinase B (AKT) signaling pathway and the vascular endothelial-derived growth factor (VEGF) signaling pathway. Further animal experimental validation showed hirudin decreased the expression of caspase-3 related to apoptosis, and inhibited the PI3K-AKT signaling pathway in the kidney of DN rats. These outcomes establish a groundwork for prospective molecular biological investigations into hirudin’s efficacy in treating DN [86].

VEGF is a key mitogen of glomerular endothelial cells (GEC), and regulates its survival, proliferation, and angiogenesis. Increased expression of VEGF in the kidney has been implicated in the pathogenesis of DN [87]. Hypoxia-inducible factor 1 (HIF-1) is a heterodimeric transcription factor that mediates cellular responses to low oxygen through the transcriptional activation of specific genes including VEGF [88]. It has been reported that hirudin significantly improved renal function in the rat model of DN, and significantly down-regulated the expression of fibronectin (FN), type IV collagen, HIF-1α, and VEGF proteins. In vitro, hirudin could reduce the expression of ECM-associated proteins in HK-2 cells treated with high glucose (HG) [72]. The group also found that hirudin significantly inhibited the migration and angiogenesis of GEC, and reversed HG-induced expression of VEGF [74]. Liposomes are recognized as efficient kidney-targeted drug delivery systems [89]. Recently, a study reported that the hirudin/liposome complex significantly increased the accumulation of hirudin in the kidney, improved renal function, and alleviated renal pathological damage in DN rat models by inhibiting the expression of VEGF [71], indicating modification or preparation of hirudin may be helpful for further application and research.

Mitogen-activated protein kinases (MAPKs) are serine/threonine kinases that convert extracellular stimulation into a variety of cellular events, including gene expression, cell proliferation, differentiation, motility, survival, and apoptosis [90]. Hyperphosphorylation of MAPK molecules ultimately activates the nuclear factor (NF)-κB and subsequently produces inflammatory molecules [91]. The p38-MAPK and NF-κB pathways play a pivotal role in the progression of CKD involving inflammation. A study found that hirudin could inhibit inflammation and podocyte apoptosis by regulating the p38 MAPK/NF-κB pathway, ultimately resulting in the protection of renal function and reducing renal pathological changes [73]. Another study showed that hirudin has the potential to diminish microangiopathy associated with nephropathy in rats with DN by inhibiting the p38MAPK/NF-kB signaling pathway [74]. To sum up, the p38 MAPK/NF-κB signaling pathway may function as a central mediator of inflammation in diabetic nephropathy.

Gasdermin D (Gsdmd) is a key factor in lytic and highly inflammatory forms of pyroptosis, and acts as a specific substrate for inflammatory caspases [92]. Converging studies reveal that pyroptosis can occur in renal intrinsic cells, and is involved in the development of DN [93]. A study found that hirudin effectively ameliorated kidney injury in DN mice by targeting Gsdmd; hirudin also suppressed pyroptosis in primary GECs, RTECs, and bone-marrow-derived macrophages in vitro [75]. These studies suggest that hirudin may play a role in programmed death in DN.

### 4.2. Hirudin in Nephrotic Syndrome

Nephrotic syndrome (NS) is characterized by massive proteinuria, hypoalbuminemia, edema, and hyperlipidemia [94], which is the third leading etiology of ESRD, and the eighth leading cause of death in America [95,96]. Podocytes are highly specialized, terminally differentiated cells and intrinsic components of the glomerulus, as part of the glomerular filtration barrier, along with a fenestrated endothelium and glomerular basement membrane [97]. In the field of renal physiology, podocytes exhibit restricted regenerative capacity. The shedding and apoptosis of these specialized cells, irrespective of their origin, inflict harm upon the glomerular filtration barrier, inevitably resulting in the onset of proteinuria [98]. A diminished count of podocytes stands as a significant indicator of both glomerulosclerosis and impaired renal function. As a result, interventions targeting the prevention of podocyte injury or the facilitation of podocyte regeneration and restoration hold substantial promise for both clinical and economic benefits.

NS-associated proteinuria results in the leakage of coagulation regulatory proteins, including antithrombin and protein S, causing enhanced thrombin generation [99]. It has been reported that nonspecific thrombin inhibition by heparin or antithrombin (AT) could attenuate proteinuria in the models of NS induced by doxorubicin and puromycin aminonucleoside (PAN) [100,101]. Although it is generally considered detrimental, enhanced coagulation activation can also have beneficial effects. A study showed thrombin demonstrated bimodal, concentration-dependent effects in glucose-challenged podocytes, while low-concentration thrombin (50 pM) was cytoprotective, and high concentration (20 nM) exacerbated glucose-mediated cytotoxicity. Similarly, factor V Leiden (FVL) mutation, related to the low but sustained activation of thrombin, prevented hyperglycemia-induced podocyte loss in mice. Consistently, the nephroprotective effect was annulled in diabetic FVL-positive mice upon anticoagulation with hirudin [102]. In a separate investigation, it was demonstrated that targeted suppression of thrombin using hirudin resulted in decreased proteinuria in models of nephrotic syndrome induced by puromycin aminonucleoside (PAN), and in rats with human diphtheria toxin receptor expression. Thrombin was observed to coexist with a marker specific to podocytes in the glomeruli of rats. Subsequently, they ascertained that podocyte injury induced by thrombin relies on the interplay of protease-activated receptors (PAR) [79]. Overall, this information suggests that thrombin plays a role in podocyte injury in nephrosis, and suggests that hirudin may modulate the interactions between thrombin and PAR, thereby regulating podocyte injury in NS.

Under the disturbance of various stimuli, endoplasmic reticulum dysfunction and endoplasmic reticulum stress (ERS) occur, leading to large amounts of unfolded and misfolded proteins accumulating in the endoplasmic reticulum. In turn, the persistence of ERS can induce abnormal apoptosis and autophagy, thereby promoting cellular damage. An accumulating body of evidence indicates that dysfunction of the endoplasmic reticulum along with stimulation of the unfolded protein response and autophagy contributes to the development and progression of kidney disease [103]. A recent study reported that hirudin mitigated renal damage and reduced proteinuria in PAN mice. It also mitigated the disruption of cytoskeletal proteins (synaptopodin, nephrin, and podocin), activation of endoplasmic reticulum stress (ERS), and apoptosis in both PAN mice and podocytes induced by PAN. This effect was achieved through the inhibition of p38 MAPK signaling-mediated ERS [80]. All evidence supports the protective effect of hirudin against podocyte injury.

### 4.3. Hirudin in Renal Interstitial Fibrosis

The pathological process of RIF is complex and is related to the increase in ECM, renal tubular epithelial phenotypic transformation (EMT), oxidative stress, and the effects of various cytokines. Excessive ECM deposition and subsidence are important hallmarks of RIF [104]. EMT refers to the phenotypic conversion of epithelial cells to fibroblast-like cells by reducing E-cadherin expression and increasing N-cadherin expression to acquire mesenchymal morphology [105]. The proliferation of fibroblasts is a precursor to the overproduction of ECM. Oxidative stress accelerates the progression of RIF. An increase in ROS and a decrease in antioxidant enzyme activity are closely associated with the development of obstructive kidney injury [106]. In addition, ROS can act as signaling molecules to participate in intracellular signaling pathways such as NF-κB, causing RIF [107]. Early studies showed thrombin stimulated FN secretion and transforming growth factor-β (TGF-β) secretion in TEC in a dose-dependent manner, which could be inhibited by thrombin inhibitors like hirudin [108]. Recently, multiple studies reported that hirudin has a significant anti-fibrotic effect on renal interstitial fibrosis.

TGF-β_1_ could induce renal fibrosis by activating canonical (Smad-based) and non-canonical (non-Smad-based) signaling pathways. In the canonical TGF-β_1_ signaling pathway, TGF-β_1_ activates the phosphorylation of Smad2 and Smad3 by the TGF-β receptor 1; then, Smad4 binds to activated Smad2/3, translocates to the nucleus, and transcribes some specific genes, which ultimately leads to activation of myofibroblasts, overproduction of ECM, and inhibited the degradation of ECM [109]. Therefore, inhibiting the TGF-β_1_ signaling pathway may be an effective solution for RIF. Recently, Yang et al. confirmed that hirudin improves kidney injury and suppresses the inflammatory response and ECM accumulation in unilateral ureteral obstruction (UUO) rats, which may be associated with the inhibition of TGF-β_1_/Smad and NF-κB signaling [76]. Xie et al. found that hirudin reduced EMT, inflammatory infiltration, and apoptosis in UUO rats and TGF-β-induced RTECs [110].

**Figure 2:**
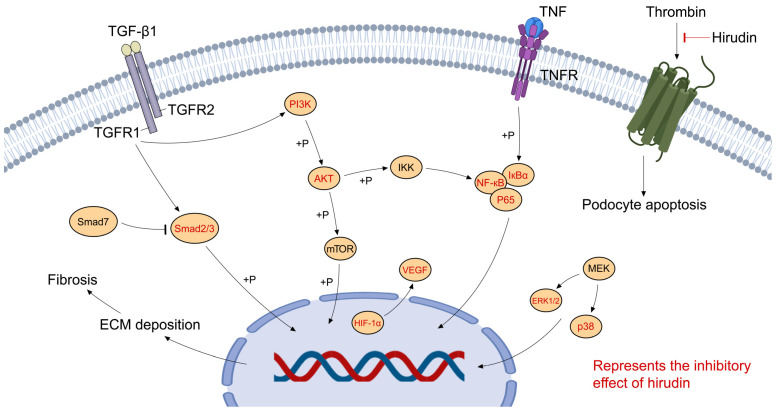
Molecular pathways in the pharmacological activities of hirudin in CKD.

Non-Smad pathways in TGF-β signaling include the phosphatidylinositol 3-kinase (PI3K)/AKT signaling pathway [111], which is involved in the process of cell proliferation, differentiation, and apoptosis [112]. PI3K/AKT signaling has been observed to correlate with ECM accumulation and EMT. Therefore, it may be involved in renal interstitial fibrosis [113]. Yu et al. found hirudin mitigated RIF and downregulated expressions of fibrosis-associated genes in UUO rats. The following transcriptomic analysis and experiments in vivo and in vitro showed hirudin significantly inhibited the PI3K/AKT signaling pathway [78]. Another study also found that hirudin suppressed the immigration and EMT of RTECs, probably by inhibiting the PI3K/AKT signal pathway [77]. The molecular pathways involved in the pharmacological activities of hirudin in CKD are shown in Figure 2.

### 4.4. Hirudin in Other Renal Disorders

#### 4.4.1. Acute Kidney Injury

Renal ischemia/reperfusion injury (IRI) is the major cause of acute kidney injury, which commonly occurs in the setting of major surgery, trauma, and sepsis [114]. IRI manifests as a sequential process involving the initial constriction of blood flow to the kidneys followed by subsequent reestablishment of blood circulation. The extent of injury induced by IRI is contingent upon the metabolic requisites of the tissue, the duration of the ischemic event, and the manner and degree of reperfusion. Prolonged periods of ischemia may lead to the demise of renal cells, compromised recovery of renal function, and the emergence of interstitial fibrosis [115]. An early study found that increased tissue factor (TF) activity during renal IR led to increased chemokine expression and subsequent neutrophil-mediated tubular injury, which was predominantly caused by thrombin-dependent PAR-1 signaling. Hirudin-treated mice and PAR-1-/- mice were protected from IRI, while treating PAR-1-/- mice with hirudin manifested no additional benefit [81]. Thus, hirudin may protect the kidney from IRI by inhibiting the interactions between thrombin and PAR-1.

#### 4.4.2. Metastatic Kidney Cancer

Once the metastatic cascade initiates and tumor cells commence colonization of distant organs, the prospect of curing cancer becomes exceedingly slim. The onset of metastasis is triggered by the release of tumor cells from the primary neoplasm, enabling infiltration into the bloodstream. This orchestrated progression follows a specific genetic sequence, namely epithelial–mesenchymal transition, facilitating the detachment of tumor cells from the primary tumor tissue and their invasion into the adjacent tumor stroma as individual cells [116]. Adhesion pathways that regulate F-actin and stress fiber assembly are commonly heightened in metastatic tumor cells, and suppressing them has been documented to possess anti-metastatic attributes [117]. A study reported that F-actin promotes colony formation of primary renal tumor cells in patients with renal metastases. Meanwhile, they found that the thrombin inhibitor hirudin inhibited F-actin formation and significantly reduced the metastatic growth of renal tumor cells in nude mice [82].

#### 4.4.3. Arteriovenous Fistula Stenosis

Hemodialysis (HD) is a widely used form of renal replacement treatment that purifies the blood by removing extra water, waste products, and harmful electrolytes from the blood [118]. The arteriovenous fistula (AVF) is the preferred vascular access for the dialysis process, providing sufficient blood to ensure adequate dialysis treatment [119]. The AVF is involved in vascular proliferation during maturation and requires dilational remodeling, including vascular smooth muscle cells (VSMC) and vascular endothelial cells [120].

As reported, M1/M2 macrophage homeostasis is involved in the renal microenvironment, and is related to AVF wall thickening and AVF maturation [121,122]. A study found that hirudin alleviated renal function and vascular injury in chronic renal failure rats induced by adenine, promoted SMC hyperplasia, and stimulated the differentiation of M1 macrophages towards M2 macrophages. In vitro, hirudin promoted the proliferation of rat VSMCs and improved the inflammatory response and apoptosis during co-incubation in an M1-conditioned medium [83].

Belonging to the serine/threonine kinase family, Rho kinase (ROCK) holds significance as a downstream effector of the small GTP-binding protein RhoA. The signaling pathway of RhoA/ROCK plays a crucial role in governing endothelial cell apoptosis and permeability [123]. In the presence of CRF serum, hirudin demonstrated the restoration of cell viability, telomere length, and telomerase activity. Furthermore, it exhibited the suppression of endothelial cell permeability and apoptosis in human umbilical vein endothelial cells. This effect is likely reliant on the inactivation of the RhoA/ROCK signaling pathway [84].

#### 4.4.4. IgA Nephropathy

IgA Nephropathy (IgAN) is the most common primary glomerular disease in the world and a significant cause of ESRD, which is characterized by massive mesangial deposition of IgA-immune complexes and mesangial expansion [124]. Imbalanced T cell function and secretion of pro-inflammatory factors are involved in the development of IgAN [125]. Hirudin improved renal function in IgAN rat models induced by oral and intravenous immunization with bovine gamma-globulin (BGG). In addition, hirudin reduced cell apoptosis, renal fibrosis, and inflammatory infiltration, and stabilized the balance of T cells [85].

## 5. Adverse Effects of Hirudin

### 5.1. Bleeding and Anticoagulant Monitoring

Bleeding stands as the foremost adverse event associated with hirudin usage. Major bleeding occurred in patients receiving lepirudin during therapy for HIT, with five hemorrhagic deaths (2.5%) in the prospective study [126]. Thus, hirudin is contraindicated in patients with active bleeding/irreversible coagulopathy. Monitoring of coagulation parameters is essential to ensure that the dose of hirudin remains within the therapeutic range. When employing activated partial thromboplastin time (APTT) monitoring, the objective is to achieve a target range of 1.5–2.5 times the baseline APTT value (generally, the mean of the laboratory normal range) [127]. Another important problem is that of renal insufficiency in patients receiving lepirudin. The half-life of hirudin rises dramatically with azotemia, which requires more frequent monitoring of APTT.

### 5.2. Immunization and Anaphylaxis

Hirudins exhibit significant immunogenicity. According to time-to-event analyses, the formation of antihirudin antibodies typically occurs within 1 to 4 weeks following the initiation of treatment with rH. Immunization rates appear consistent between lepirudin and desirudin. Certain antibodies lack neutralizing and precipitating properties, potentially resulting in prolonged drug circulation and increased APTT levels [128].

Some individuals may experience allergic reactions to hirudin, while a rash is the most common allergy symptom. Greinacher reported that four patients experienced fatal anaphylaxis after lepirudin injections, and died quickly after the onset of the event [129]. Risk factors associated with anaphylactic reactions to hirudin may involve factors such as the initial administration of a bolus dose, intravenous administration instead of subcutaneous, prolongation of therapy beyond 3 days in a single course, and re-administration of lepirudin within a 100-day timeframe [130].

### 5.3. Drug Interactions

Hirudin may interact with other medications, particularly those that affect blood clotting or are metabolized by the kidneys. This can potentially lead to an increased risk of side effects or reduced effectiveness of either medication. Hirudin is prohibited from being used in combination with sulodexide because sulodexide is a heparinoid, and combined use will increase the efficacy of the drug [131].

## 6. Prospects and Conclusions

CKD is a common public health epidemic associated with high morbidity and mortality. Hirudin, an active compound from natural products, has shown protective effects in various models of CKD. However, most research reports focused on observations or phenotypic changes rather than mechanisms of action associated with key pathogenesis. Based on the existing research, we found that the protective mechanism of hirudin on the kidney is mainly to inhibit the inflammatory response and thrombin. However, few clinical studies have been carried out so far, indicating that the clinical application of hirudin in kidney disease still needs further research. The low productivity and low oral bioavailability of hirudin may restrict its clinic application. rH or the drug delivery system with hirudin may bring this historic natural compound to the forefront of therapeutic agents for the treatment of CKD.

## Figures and Tables

**Table 1:** Pharmacologic properties and approved indications of hirudin derivatives.

Derivatives	Lepirudin	Desirudin	Bivalirudin
Indication	Treatment of thrombosis complicating HIT	Thrombosis prophylaxis after hip or knee arthroplasty	Patients with UA undergoing PTCA; PCI with temporary use of GPI; patients at risk of HIT undergoing PCI
Thrombin affinity (Ki)	0.23 pM	0.26 pM	1.9 nM
Route of administration	Intravenous, subcutaneous	Intravenous, subcutaneous	Intravenous
Plasma half-life	80 min	60 min	25 min
Clearance	Renal	Renal	Proteolysis (80%)

**Table 2:** Hirudin in the treatment of kidney diseases.

Kidney Diseases	Functions	References
Diabetic nephrology	Inhibiting the HIF-1α/VEGF signaling pathway;	[71,72]
	Inhibiting the p38 MAPK/NF-κB signaling pathway;	[73,74]
	Inhibiting Gsdmd-mediated pyroptosis	[75]
Renal interstitial fibrosis	Inhibiting the TGF-β1/Smad and NF-κB signaling pathways;	[76]
	Inhibiting the PI3K/AKT signaling pathways	[77,78]
Nephrotic syndrome	Inhibiting the interactions between thrombin and PAR;	[79]
	Inhibiting p38 MAPK signaling-mediated ERS	[80]
Acute kidney injury	Inhibiting the interactions between thrombin and PAR	[81]
Metastatic kidney cancer	inhibiting F-actin formation and reducing metastatic growth	[82]
Arteriovenous fistula stenosis	Promoting SMC hyperplasia; stimulating the differentiation of M1 macrophage towards M2 macrophage Regulates endothelial cell permeability and apoptosis, inhibiting RhoA/ROCK signaling	[83,84]
IgA nephrology	Reducing cell apoptosis; inflammatory response and keeping balance of T cells	[85]

## Data Availability

Not applicable.
